# Exposure to Nature Sounds through a Mobile Application in Daily Life: Effects on Learning Performance among University Students

**DOI:** 10.3390/ijerph192114583

**Published:** 2022-11-07

**Authors:** Jiutong Luo, Minhong Wang, Boyin Chen, Meng Sun

**Affiliations:** 1 Advanced Innovation Center for Future Education, Faculty of Education, Beijing Normal University, Beijing 100875, China; 2 Center for Educational Science and Technology, Beijing Normal University, Zhuhai 519087, China; 3 Health Science Center, Shenzhen University, Shenzhen 518060, China; 4 Faculty of Education, The University of Hong Kong, Hong Kong; 5 Department of Educational Information Technology, East China Normal University, Shanghai 200062, China; 6 College of Education for the Future, Beijing Normal University, Zhuhai 519087, China

**Keywords:** exposure to nature sounds, mobile application, learning performance, university students

## Abstract

Previous studies have revealed the restorative effects of exposure to natural environments on psychological well-being and cognitive performance. Recent studies have reported the effects of exposure to nature sounds (e.g., the sounds of birds, rainfall, and waves) through a mobile application on reducing students’ mental fatigue and improving their cognitive performance. However, it remains unknown whether exposure to nature sounds through a mobile application may influence students’ learning performance. To address the gap, we conducted a study with 71 university students. During the four-week intervention, 36 students in the experimental group were exposed to nature sounds through a free mobile application for at least 30 consecutive minutes per day when working on academic-related tasks; 35 students in the control group did not have such exposure when working on similar tasks. The results show that students in the experimental group outperformed those in the control group in their engagement in deep learning, frequency of academic procrastination, and academic self-efficacy. The findings reveal the promising effects of exposure to nature sounds through a mobile application on improving students’ learning performance. The implications of the findings are discussed.

## 1. Introduction

Many university students are nowadays suffering from mental fatigue and stress caused by busy and competitive daily activities [1,2], learning with highly cognitive demanding tasks [3,4], and extensive use of digital devices in their daily work [5,6,7,8,9,10]. Research shows that exposure to natural environments has substantial benefits for improving psychological well-being (e.g., positive effect) and cognitive performance (e.g., working memory) [11,12,13,14,15,16], which have the potential to improve learning performance. A prior study suggested that exposure to nature sounds (e.g., the sounds of birds, rainfall, and waves) through a mobile application in daily life can reduce university students’ mental fatigue and improve their cognitive performance [17]. However, research on whether exposure to nature sounds in daily life may influence students’ learning performance is underdeveloped. Therefore, this study aimed to fill the gap by examining the benefits of exposure to nature sounds through a mobile application in the daily lives of university students’ learning performances.

### 1.1. Impact of Exposure to Nature (Including Nature Sounds)

The benefits of exposure to natural environments on psychological and cognitive aspects have been extensively investigated by researchers [11,12,13,14,15,16]. According to the literature, the psychological and cognitive effects of exposure to nature have been supported by two widely recognized theories, i.e., stress recovery theory [18,19] and attention restoration theory [20,21], and were examined in many empirical studies [16,22,23,24,25].

Empirical studies have reported the restorative effects of exposure to natural environments (including nature sounds) in psychological and cognitive dimensions. In terms of the *psychological* dimension, exposure to nature can promote positive psychological well-being and reduce stress [25,26,27]. For example, Hamann and Ivtzan [25] reported that exposure to a natural environment for 30 min per day for one month could improve the positive effects (e.g., happiness, meaning in life, and mindfulness) and decrease the negative effects (e.g., distress and upset). In terms of the *cognitive* dimension, exposure to nature can improve attention and executive function [17,28,29] and boost the flow state [17,30,31]. For example, presenting nature scenes (e.g., Hawaiian beach) through virtual reality in an open office helped office workers to foster a flow state [30], which is a positive experiential and mental state highly related to cognitive performance occurring when people are completely immersed in their activities [32]. Although exposure to nature has shown significant impacts on improving psychological and cognitive performances, which may influence learning performance, it remains unknown whether exposure to nature may affect learning performance [33,34,35].

Regarding exposure to nature, researchers have further explored the benefits of exposure to visual elements (e.g., natural scenery pictures) and auditory stimuli (e.g., nature sounds) of natural environments [23,36,37]. For example, a 40-s view of a picture of a flowering meadow with a green roof was found to be sufficient to boost sustained attention [36]. Nature sounds (e.g., the sounds of birds, rainfall, and waves) are perceived as pleasant and relaxing and, thus, they could reduce mental stress and physical tension (e.g., muscle tension, pulse rate) and improve working memory [1,27,29]. In general, the potential restorative effects of nature sounds on physiological, psychological, and cognitive aspects have gained increasing attention [37].

### 1.2. Potential Impact of Exposure to Nature (Including Nature Sounds) on Learning

Exposure to natural environments is related to positive learning outcomes in educational settings [38,39,40]. Research shows that greenness (i.e., the density of trees or the amount of vegetation) within or surrounding the schoolyard is positively related to student learning performances [40,41,42,43]. A prior study reported that tree cover density within a 1-mile radius of a school is positively associated with the academic performances of students, as reflected by American College Test (ACT) scores and colleague readiness [43]. Further, researchers revealed that exposure to green spaces can improve attentional functioning and help students recover from stress and mental fatigue [15,44,45]. These studies indicated the potential causal relationship between exposure to natural environments and student learning performances, which, however, has not yet been fully verified.

Existing findings on the effects of exposure to nature on psychological and cognitive performances provide some insights into the possible effects of exposure to nature (including nature sounds) on learning performance. *First*, exposure to natural environments, including nature sounds, could help people reach a flow state [17,30,31], which might affect learning performance. The flow state is a positive experiential and mental state related to a high level of engagement or complete immersion in certain activities [32]. Research shows that it is important to help students improve their learning performances through deep learning, i.e., a high level of engagement in learning to maximize the understanding of learning materials and identify the underlying meaning of learning tasks [46,47,48,49]. Therefore, this study examined the possible effects of exposure to nature sounds on students engaging in deep learning, an important measure of learning performances among university students.

*Second*, the benefits of exposure to nature have also been reported as improving attention and executive functions [17,28,29], which might affect learning performance. In particular, the attention restoration theory places special emphasis on directed attention [20,21], which is a resource for self-regulation [50]. Empirical studies found that exposure to nature can improve self-regulation among different age groups [51,52,53]. In the academic context, self-regulation failure could lead to academic procrastination [54,55], which refers to voluntarily delaying academic tasks despite expecting to be worse off [56]. Due to the increased mental fatigue among university students, academic procrastination has become an emerging problem and common phenomenon [57]. Therefore, this study examined the possible effects of exposure to nature sounds on academic procrastination, an important measure of learning performance among university students.

*Third*, exposure to nature can promote positive psychological well-being and reduce stress [25,26,27], which might lead to a positive effect on effective learning. For example, using a mobile application to listen to nature sounds for four weeks significantly increases university students’ positive emotions [17]. Garden-based learning can improve students’ self-esteem and self-confidence in learning [58]. Thus, exposure to nature can affect psychological indicators directly relevant to learning. In particular, academic self-efficacy, which refers to students’ beliefs about their abilities to achieve educational goals [59,60], is among the most significant predictors of university students’ academic success [61]. Therefore, this study examined the possible effects of exposure to nature sounds on academic self-efficacy, an important measure of learning performance among university students.

### 1.3. The Present Study

Previous studies have revealed the restorative effects of exposure to natural environments on psychological well-being (e.g., positive effect) and cognitive performance (e.g., working memory), which have the potential to affect learning performance. A prior study suggested that exposure to nature sounds (e.g., the sounds of birds, rainfall, and waves) through a mobile application in daily life can reduce university students’ mental fatigue and improve their cognitive performance [17]. However, it remains unknown whether exposure to nature sounds through a mobile application in daily life may influence students’ learning performance. To address the gap, this study investigated the benefits of exposure to nature sounds through a mobile application in the daily lives of students’ learning performances in terms of student engagement in deep learning, academic procrastination, and academic self-efficacy. The following research questions (RQs) were explored in this study:

RQ1: Does exposure to nature sounds through a mobile application in daily life impact students’ engagement in deep learning?

RQ2: Does exposure to nature sounds through a mobile application in daily life impact students’ academic procrastination?

RQ3: Does exposure to nature sounds through a mobile application in daily life impact students’ academic self-efficacy?

## 2. Method

### 2.1. Participants

The participants were 71 university students from a university located in a highly competitive city in southeast China. The competitive urban context, busy studying schedules, as well as peer competition regarding academic achievements, internship positions, and social life, have directly affected university students’ daily lives. They are often busy attending lectures, working on projects, and participating in academic and social events, with limited time for exposure to nature. Moreover, they have experienced external pressures in peer competition and job-seeking practices, making them feel mentally exhausted or mental fatigued [17]. The study received ethical approval from the Human Research Ethics Committee of the researchers’ university. All participants signed a written consent form to participate in the study.

Following the prior power calculation, a sample of 52 participants was needed to achieve a medium effect size (Cohen’s ƒ = 0.20) with a power of 0.80, alpha of 0.05 (two-tailed), and correlations of 0.50 among repeated measures.

The participants were recruited through an advertisement posted on a social media platform, with the purpose to approach more participants from diverse backgrounds. We first received 76 responses and used the SPSS function “random sample of cases” to select 50% of cases from the 1–76 numbering list. The selected participants were assigned to the experimental group and the rest were assigned to the control group. Five participants who did not finish the study were excluded from the study. There were 36 students (16 males and 21 females) in the experimental group and 35 students (13 males and 22 females) in the control group. No gender difference was found between the two groups (χ^2^ = 0.15, *p* = 0.70). The mean age of participants was 19.15 years (SD = 1.24).

### 2.2. Measures

#### 2.2.1. Engagement in Deep Learning

To measure student engagement in deep learning, the deep learning process scale from the Study Process Questionnaire proposed by Biggs et al. [48] was adopted. It included 10 items, such as “I find that I have to do enough work on a topic so that I can form my own conclusions before I am satisfied”; “I find most new topics interesting and often spend extra time trying to obtain more information about them”; and “I test myself on important topics until I understand them completely”. Participants were asked to respond to these items on a 5-point Likert scale (from 1 = ‘rarely true of me’ to 5 = ‘almost always true of me’). Cronbach’s α of the deep learning process scale for the pre- and post-surveys were 0.70 and 0.76, respectively.

#### 2.2.2. Academic Procrastination

The academic procrastination scale was adopted from the Academic Procrastination State Inventory [62]. It included 13 items that measured how often students “drift off into daydreams while studying,” or “put off the completion of a task” in their daily life. All items were measured on a 5-point Likert scale (from 1 = ‘never’ to 5 = ‘always’). The Cronbach’s α of the academic procrastination scale for the pre- and post-surveys were 0.83 and 0.89, respectively.

#### 2.2.3. Academic Self-Efficacy

To measure the participants’ academic self-efficacy, the 8-item self-efficacy for learning and performance scale from the Motivated Strategies for Learning Questionnaire [63] was adopted. Participants were asked to respond to the items such as “I believe I will receive an excellent grade this semester” on a 5-point Likert scale (from 1 = ‘strongly disagree’ to 5 = ‘strongly agree’). Cronbach’s α of the academic self-efficacy scale for the pre- and post-surveys were 0.89 and 0.91, respectively.

#### 2.2.4. Weekly Reports and Comments

Weekly reports and open-ended questions were also used to verify the findings from the above measures. *First*, this study adapted one item from a previous study [64] to measure the distraction in learning perceived by participants weekly during the experiment. Participants responded to this item (“overall, how distracted were you feel during the learning this week”) on a 6-point Likert scale (from 1 = very serious to 6 = never). *Second*, the 3-item productivity scale [64] was adapted to measure participants’ learning productivity weekly during the experiment. Participants were asked to respond to the items (e.g., “overall week, I finished the learning task that was most important to me”) on a 6-point Likert scale (from 1 = strongly disagree to 6 = strongly agree). Cronbach’s α of each learning productivity scale was 0.83, 0.74, 0.90, 0.84, and 0.87 for the four weeks, respectively. *Finally*, the participants in the experimental group were asked to comment on the use of the nature sound application at the end of the study.

### 2.3. Procedure

This study was conducted two weeks after the midterm exam and ended three weeks before the final exam. The experiment lasted for 4 weeks. Before the experiment (Week 0), all participants were given an introduction to the experiment, followed by a pre-study survey on their learning performances (in terms of engagement in deep learning, academic procrastination, and academic self-efficacy) and their demographic information. They also completed a survey on perceived distraction and learning productivity, as part of the weekly reports.

From Week 1 to Week 4, the students assigned to the experimental group were asked to use a free mobile application for exposure to nature sounds for at least 30 min per day when they worked on academic-related tasks. The nature sounds used in this study included computer-simulated sounds of birds, rainfall, waves, and the wind, among others, which are common types of nature sounds, not region-specific. During the experiment, each student could freely switch between the above-mentioned types of sounds at any time during the experiment according to their own interests. Students used the mobile application individually with earphones while they studied in a library or a study room. They often studied at a library or a study room to finish their coursework in different subjects. The students in the control group were asked to learn in a traditional way without any intervention. In addition, students in both groups were asked to complete a short weekly survey on perceived distraction and learning productivity.

At the end of Week 4, a post-study survey was administrated to all students to measure their learning performances (in terms of engagement in deep learning, academic procrastination, and academic self-efficacy) at the end of the study. Students in the experimental group were asked to comment on the use of the nature sound application during their study.

### 2.4. Data Analysis

The mixed analysis of variances (ANOVAs) was conducted to examine the differences in students’ engagement in deep learning, frequency of academic procrastination, and academic self-efficacy between the two conditions (i.e., experimental and control groups) and two times (i.e., pre-test and post-test). The mixed ANOVAs could compare the differences between the two groups as well as detect the trends from the pre-study to the post-study. Moreover, the repeated measures ANOVAs were used to analyze students’ perceived distractions and learning productivities over the five time points (from Week 0 to week 4). Finally, the students’ comments (regarding using the nature sound application) were summarized to verify the findings.

## 3. Results

Table 1 presents the descriptive statistics of the students’ learning performances in terms of student engagement in deep learning, academic procrastination, and academic self-efficacy. The comparisons of student performances between the two groups are presented as follows.

### 3.1. Engagement in Deep Learning

**Figure 1:**
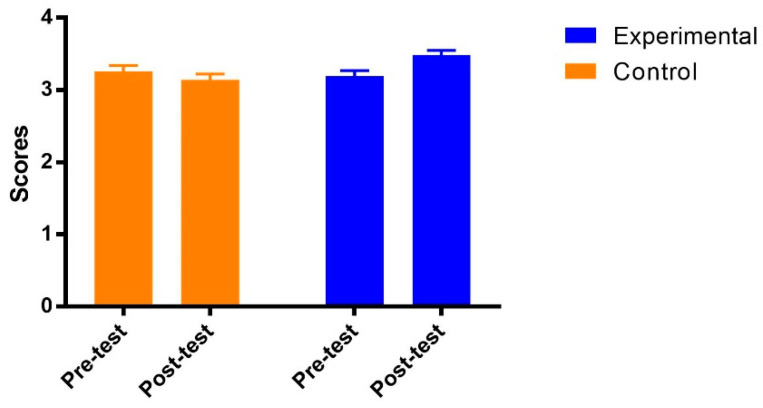
Adjusted means (SEs) for deep learning by group and test.

The results of mixed ANOVA on student engagement in deep learning showed no main effects of both groups [F(1, 69) = 1.90, *p* = 0.17 > 0.05, η_p_^2^ = 0.03] and test time [F(1, 69) = 3.50, *p* = 0.07 > 0.05, η_p_^2^ = 0.05]. The interaction between the group and test time was significant [F(1, 69) = 20.33, *p* < 0. 001, η_p_^2^ = 0.23]. As shown in Figure 1, the post hoc analysis indicated that student engagement in deep learning in the experimental group was significantly improved from the pre-test to post-test [Adjusted Mean_pre_ (SE) = 3.19 (0.08); Adjusted Mean_post_ (SE) = 3.48 (0.07), *p* = 0.000] with a large effect size (Cohen’s ƒ = 0.55). However, student engagement in deep learning in the control group remained stable from the pre-test to post-test [Adjusted Mean_pre_ (SE) = 3.26 (0.08); Adjusted Mean_post_ (SE) = 3.14 (0.08), *p* = 0.07].

### 3.2. Academic Procrastination

**Figure 2:**
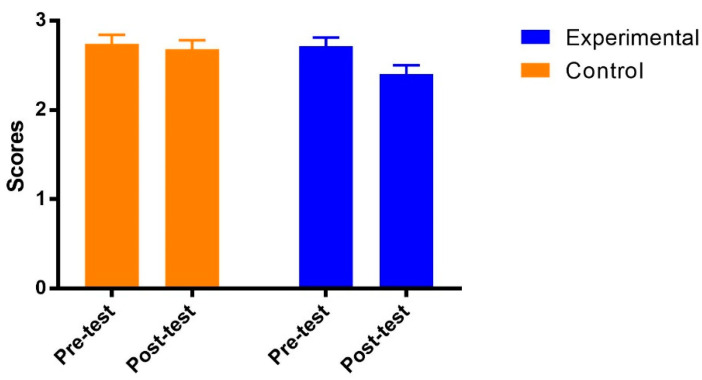
Adjusted means (SEs) for academic procrastination by group and test.

The results of mixed ANOVA on academic procrastination showed no main effect of the group [F(1, 69) = 1.34, *p* = 0.25 > 0.05, η_p_^2^ = 0.02], but the effect of test time [F(1, 69) = 11.81, *p* = 0.001 < 0.01, η_p_^2^ = 0.15] was significant. The interaction between the group and test time was significant [F(1, 69) = 5.80, *p* = 0.02 < 0. 05, η_p_^2^ = 0.08]. As shown in Figure 2, the post hoc analysis indicated that the academic procrastination of students in the control group remained stable from the pre-test to post-test [Adjusted Mean_pre_ (SE) = 2.74 (0.10); Adjusted Mean_post_ (SE) = 2.68 (0.10), *p* = 0.47]. However, academic procrastination of those in the experimental group was significantly decreased from the pre-test to post-test [Adjusted Mean_pre_ (SE) = 2.71 (0.10); Adjusted Mean_post_ (SE) = 2.40 (0.10), *p* = 0.000] with a medium effect size (Cohen’s ƒ = 0.29).

### 3.3. Academic Self-Efficacy

**Figure 3:**
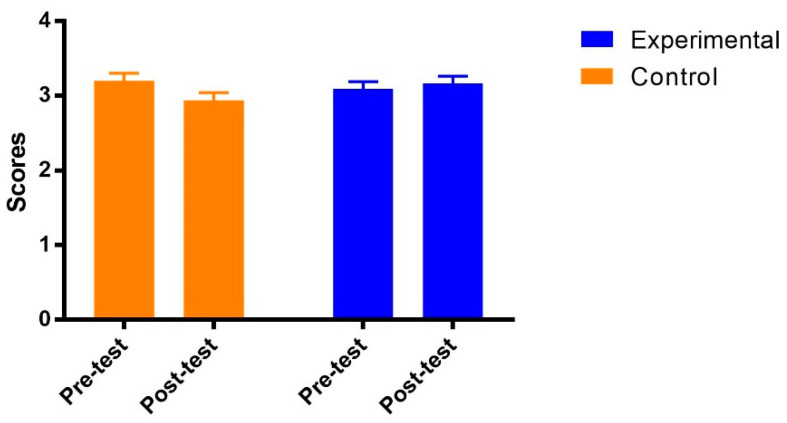
Adjusted means (SEs) for academic self-efficacy by group and test.

The results of mixed ANOVA on academic self-efficacy showed no main effects of both groups [F(1, 69) = 2.25, *p* = 0.14 > 0.05, η_p_^2^ = 0.03] and test time [F(1, 69) = 0.19, *p* = 0.66 > 0.05, η_p_^2^ = 0.00]. The interaction between the group and test time was significant [F(1, 69) = 7.10, *p* = 0.01 < 0. 05, η_p_^2^ = 0.09]. As shown in Figure 3, the post hoc analysis indicated that the academic self-efficacy of students in the experimental group remained stable from the pre-test to post-test [Adjusted Mean_pre_ (SE) = 3.09 (0.10); Adjusted Mean_post_ (SE) = 3.16 (0.10), *p* = 0.41]. However, academic self-efficacy in the control group was significantly decreased from the pre-test to post-test [Adjusted Mean_pre_ (SE) = 3.20 (0.10); Adjusted Mean_post_ (SE) = 2.94 (0.10), *p* = 0.005] with a large effect size (Cohen’s ƒ = 0.31).

### 3.4. Weekly Reports and Students’ Perceptions

**Figure 4:**
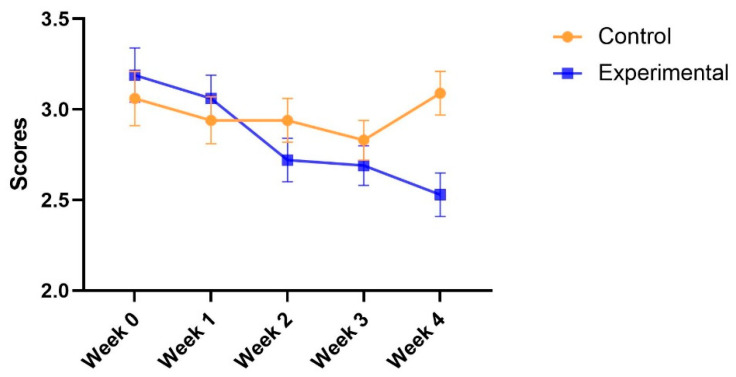
Adjusted means (SEs) for perceived distraction by group and test.

**Perceived distraction.** Regarding perceived distraction, Mauchly’s test of sphericity (*p* = 0.71) was not significant; therefore, the sphericity was assumed. The results indicated that there was no main effect of the group [F(1, 69) = 1.55, *p* = 0.22 > 0.05, η_p_^2^ = 0.02], but the main effect of test time was significant [F(4, 276) = 3.59, *p* = 0.007 < 0.01, η_p_^2^ = 0.05]. The interaction between the group and test time was significant [F(4, 276) = 1.43, *p* = 0.016 < 0.05, η_p_^2^ = 0.04]. The post hoc analysis shown in Table 2 indicated that perceived distraction remained stable in the control group, but decreased in the experimental group. Figure 4 presents the changes.

**Figure 5:**
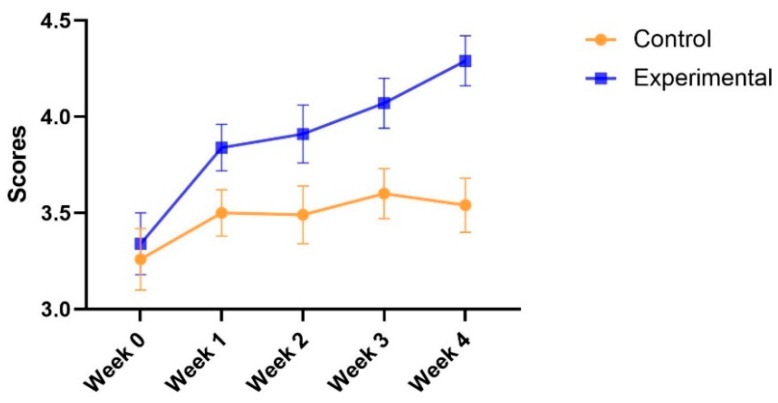
Adjusted means (SEs) for learning productivity by group and test.

**Learning productivity.** With respect to learning productivity, Mauchly’s test of sphericity (*p* = 0.000) was significant; therefore, sphericity was not assumed. Meanwhile, the epsilon was over 0.75. In this case, the results of Huynh–Feldt correction were reported. The results indicated that there were main effects of both groups [F(1, 69) = 10.94, *p* = 0.001 < 0.01, η_p_^2^ = 0.14] and test time [F(3.598, 248.252) = 7.964, *p* = 0.000 < 0.001, η_p_^2^ = 0.10]. The interaction between the group and test time was not significant [F(3.598, 248.252) = 1.99, *p* = 0.10 > 0.05, η_p_^2^ = 0.03]. The results are presented in Figure 5.

The post hoc analysis indicated that the experimental group (Adjusted mean = 3.89, SE = 0.09) reported a higher level of learning productivity than the control group (Adjusted mean = 3.48, SE = 0.09). The means (SDs) of the learning productivities for the four weeks (Weeks 1–4) were 3.30 (0.11), 3.67 (0.09), 3.70 (0.10), 3.84 (0.90), and 3.92 (0.10), respectively. The results showed that the learning productivity at week 0 was lower than those of the other four times (*p* < 0.01 for all) and learning productivity at week 1 was also lower than that of week 4 (*p* < 0.05). Moreover, the *t*-test showed that there was no difference in learning productivity between the two groups at the beginning of the study [t (69) = 0.38, *p* = 0.70 > 0.05]. However, there were consistent differences between the two groups in the following weeks [t (69) > 2.02, *p* < 0.05 for all]. The results are shown in Table 3.

**Student comments.** Students’ comments on using the mobile application for exposure to nature sounds during the study could be summarized in three aspects. *First*, nature sounds can eliminate environmental distractions. For example, some participants (Students 4, 9, 19, and 25) mentioned that listening to nature sounds from the mobile application could block out noises or other interferences (e.g., smartphone notifications) from their surroundings, and this allowed them to concentrate on learning in a calm environment. *Second*, nature sounds are helpful for psychological well-being. Some participants (Students 3, 16, 17, and 23) commented that listening to nature sounds could let them easily calm down, make them feel relaxed, and reduce their anxiety while studying. *Third*, nature sounds could help them get into a flow state while studying. Some participants (Students 2, 4, 6, and 22) mentioned that when listening to nature sounds, they could easily immerse themselves into a task and even lost track of time. Some students (e.g., Student 26) commented that they did not realize that they had learned for such a long time.

## 4. Discussion

The results of this study show that after the 4-week exposure to nature sounds through a mobile application, students in the experimental group outperformed their counterparts in the control group on learning performances in terms of engagement in deep learning, academic procrastination, and academic self-efficacy. The details are discussed as follows.

### 4.1. Engagement in Deep Learning

The survey results revealed that students with exposure to nature sounds through a mobile application improved their engagement in deep learning from the beginning to the end of the study, which was not found in their counterparts in the control group. The weekly reports indicated decreased distraction from the beginning to the end of the study perceived by the students with exposure to nature sounds, which was not found in the control group. Students’ comments also indicated that nature sounds can help them get into a flow state during their study. This finding is consistent with previous studies, which suggest that exposure to natural environments, including nature sounds, helped students reach an immersed flow state [17,30,31]. Researchers have suggested that students who achieve the flow state would be more motivated to carry out further learning activities [65]. They will easily engage in deep learning by immersing themselves in tasks that give them a feeling of satisfaction; they can work hard before drawing any conclusions and spend extra time to obtain more relevant information about learning topics [48]. Therefore, exposure to nature sounds through a mobile application could benefit student engagement in deep learning.

### 4.2. Academic Procrastination

The survey results revealed that the academic procrastination of the students using the nature sound application decreased from the beginning to the end of the study, which was not found in the control group. The weekly reports showed that the students with exposure to nature sounds had a higher level of learning productivity than those without exposure. A possible reason could be that students using the nature sound application improved their self-regulation, which could reduce their academic procrastination. As suggested by the literature, exposure to nature could benefit self-regulation among different age groups [51,52,53] through three different pathways [53], i.e., promoting children’s play outside, protecting against risk factors, and restoration from cognitive depletion. Similarly, in our case, the finding could be explained in the following three aspects. *First*, using the nature sounds application while studying in daily life might foster students to improve their study habits and become more self-regulated with their studies. *Second*, exposure to nature sounds through a mobile application might protect students against distractions from the surrounding environment. Student comments indicated that nature sounds can eliminate environmental distractions. In this way, students could concentrate more on learning and finishing tasks on time and, thus, reduce academic procrastination. *Third*, exposure to nature sounds helps to restore cognitive depletion, in particular, directed attention [20,21], which is a resource for self-regulation [50]. Overall, the result suggests that exposure to nature sounds through a mobile application has the potential to decrease academic procrastination, which is a phenomenon among university students that has received increased attention [57].

### 4.3. Academic Self-Efficacy

The survey results indicated that students with exposure to nature sounds through a mobile application maintained their academic self-efficacy during the study, while those in the control group decreased their academic self-efficacy during the experiment. The result is supported by the students’ weekly reports showing that students who used the nature sound application had a higher level of learning productivity than those who did not use the nature sound application. This finding is consistent with previous studies that suggest that the impact of exposure to nature on improving self-confidence, self-esteem [58], and cognitive performance [1,27,29] are highly related to self-efficacy. Nowadays, with the increased demand for students to deal with real-world problems in complex dynamic environments [66], many students suffer from fatigue or burnout, making it difficult for them to develop and maintain self-efficacy [67]. Exposure to nature sounds through a mobile application has the potential to help students maintain self-efficacy by improving their self-confidence and cognitive performance.

## 5. Conclusions

This study examined the benefits of exposure to nature sounds through a mobile application on university students’ learning performance. The results show that students in the experimental group outperformed those in the control group in terms of their engagement in deep learning, frequency of academic procrastination, and self-efficacy. While previous research studies have reported on the positive effects of exposure to natural environments, including nature sounds on psychological well-being and cognitive performance, the findings of this study shed light on the promising effects of exposure to nature sounds on improving the learning performances among university students.

The findings have two implications. First, this study extends the theoretical assumptions of stress recovery theory and attention restoration theory to academic learning. While previous studies have suggested an underlying link between exposure to nature and academic performance, this study moves one step further to reveal the direct link between exposure to nature sounds and learning performance. *Second*, with the wide use of mobile devices and mobile applications in recent years, people (including students) have more opportunities to access nature sounds, which are important aspects of the natural environment in one’s daily life. Exposure to nature sounds through a mobile application, which has shown promising effects in improving psychological well-being and learning performances among university students, can be introduced to university students to improve their learning and well-being.

Nevertheless, this study has some limitations. *First*, this study adopted self-reported measures to assess learning performance in terms of student engagement in deep learning, academic procrastination, and academic self-efficacy. Future studies should consider employing more objective measures (e.g., test scores) to extend the investigation of learning performance. *Second*, the participants of this study were university students from one university, which may constrain the generalization of the findings to some extent. Future studies could include students in other age groups and other regions. *Third*, this study did not compare the effects of exposure to nature sounds with other contexts, i.e., a real nature environment. Future studies may consider including one more comparison group immersed in a real nature environment. *Finally*, this study did not examine how students’ individual differences (e.g., personality) might influence the effects of exposure to nature sounds through a mobile application on their learning performances. Future studies are needed to address this issue.

## Figures and Tables

**Table 1:** Descriptive statistics of all variables for both groups.

Measures	Group	Pre-Test		Post-Test	
		Mean	SD	Mean	SD
Deep learning	Experimental group	3.19	0.09	3.48	0.08
	Control group	3.26	0.06	3.14	0.07
Academic procrastination	Experimental group	2.71	0.10	2.40	0.10
	Control group	2.74	0.10	2.68	0.11
Academic self-efficacy	Experimental group	3.09	0.10	3.16	0.09
	Control group	3.20	0.10	2.94	0.10

**Table 2:** Post hoc analyses on the interaction effects of the group and five times on the perceived distraction.

	Time	Adjusted Estimated Means (SE)	Comparisons
Experimental	W0	3.19 (0.15)	W0 = W1, W0 < W2 **, W0 < W3 **, W0 < W4 ***
	W1	3.06 (0.13)	W1 < W2 *, W1 < W3 *, W1 < W4 **
	W2	2.72 (0.12)	W2 = W3, W2 = W4
	W3	2.69 (0.11)	W3 = W4
	W4	2.53 (0.12)	–
Control	W0	3.06 (0.15)	W0 = W1, W0 = W2, W0 = W3, W0 = W4
	W1	2.94 (0.13)	W1 = W2, W1 = W3, W1 = W4
	W2	2.94 (0.12)	W2 = W3, W2 = W4
	W3	2.83 (0.11)	W3 = W4
	W4	3.09 (0.12)	–

* *p* < 0.05, ** *p* < 0.01, *** *p* < 0.001. W0 = beginning of the study, W1–4 = Week 1–4. The differences were highlighted in bold text.

**Table 3:** Results of the t-tests on learning productivity between two groups across five times.

	Week 0	Week 1	Week 2	Week 3	Week 4
Mean (SD) of experimental group	3.34 (0.90)	3.84 (0.72)	3.91 (0.94)	4.07 (0.73)	4.29 (0.81)
Mean (SD) of control group	3.26 (0.98)	3.50 (0.73)	3.49 (0.79)	3.60 (0.80)	3.54 (0.80)
t	0.38	2.02 *	2.03 *	2.62 *	3.89 ***
df	69	69	69	69	69

* *p* < 0.05, *** *p* < 0.001.

## Data Availability

The datasets generated during the current study are not publicly available due to the ethical requirements but are available from the corresponding author upon reasonable request.
